# Endocannabinoid Receptors Gene Expression in Morbidly Obese Women with Nonalcoholic Fatty Liver Disease

**DOI:** 10.1155/2014/502542

**Published:** 2014-04-23

**Authors:** Teresa Auguet, Alba Berlanga, Esther Guiu-Jurado, Ximena Terra, Salomé Martinez, Carmen Aguilar, Elisa Filiu, Ajla Alibalic, Fàtima Sabench, Mercé Hernández, Daniel Del Castillo, Cristóbal Richart

**Affiliations:** ^1^Grup GEMMAIR (AGAUR) and Grup de Recerca en Medicina Aplicada, Departament de Medicina i Cirurgia, IISPV, Hospital Universitari Joan XXIII, Universitat Rovira i Virgili (URV), Mallafré Guasch 4, Catalonia, 43007 Tarragona, Spain; ^2^Servei Medicina Interna, Department of Internal Medicine, Hospital Universitari de Tarragona Joan XXIII, Universitat Rovira i Virgili, Mallafré Guasch 4, Catalonia, 43007 Tarragona, Spain; ^3^Servei Anatomia Patològica, Hospital Universitari Joan XXIII Tarragona, Mallafré Guasch 4, Catalonia, 43007 Tarragona, Spain; ^4^Servei de Cirurgia, Hospital Sant Joan de Reus, Avenida del Dr. Josep Laporte 2, Catalonia, Tarragona, 43204 Reus, Spain

## Abstract

*Background*. Recent reports suggest a role for the endocannabinoid system in the pathology of nonalcoholic fatty liver disease (NAFLD). The aim of this study was to investigate the relationship between liver expression of cannabinoid (CB) receptor subtypes, CB1 and CB2, in morbidly obese (MO) women with different histological stages of NAFLD. *Methods*. We analysed hepatic CB1 and CB2 mRNA expression, and the expression of genes involved in lipid metabolism in 72 MO women, subclassified by liver histology into MO with normal liver (NL, *n* = 16), simple steatosis (SS, *n* = 28), and nonalcoholic steatohepatitis (NASH, *n* = 28) by enzyme-linked immunosorbent assay and RT-PCR. *Results*. We found that CB1 mRNA expression was significantly higher in NASH compared with SS and correlated negatively with PPAR**α**. Regarding CB2, CB2 mRNA expression correlated positively with ACC1, PPAR**γ**, IL6, TNF**α**, resistin, and adiponectin. *Conclusions*. The increased expression of CB1 in NASH and the negative correlation with PPAR**α** suggest a deleterious role of CB1 in NAFLD. Regarding CB2, its positive correlation with the anti-inflammatory molecule adiponectin and, paradoxically, with inflammatory genes suggests that this receptor has a dual role. Taken together, our results suggest that endocannabinoid receptors might be involved in the pathogenesis of NAFLD, a finding which justifies further study.

## 1. Introduction

Obesity, as part of the metabolic syndrome, is one of the major risk factors in the development of fatty liver [1, 2]. Nonalcoholic fatty liver disease (NAFLD) has become the most common liver disorder in developed countries, affecting over one-third of the population [3, 4]. NAFLD has frequently been associated with obesity, type 2 diabetes mellitus, hyperlipidemia, and insulin resistance [5]. The spectrum of the disease ranges from simple steatosis to steatohepatitis, a condition that associates steatosis, liver inflammation, hepatocellular injury, and activation of fibrogenic pathways with a 10–20% risk of developing cirrhosis within 10 to 20 years [6]. The transition from steatosis (SS) to nonalcoholic steatohepatitis (NASH) is not completely understood and appears multifactorial. Recent studies have revealed a role of lipotoxic fatty acid metabolites originating from the adipose tissue or from* de novo *lipogenesis in the development of hepatocellular injury [7]. Increasing evidence suggests that a fatty liver is more vulnerable to factors that lead to inflammation and fibrosis [8, 9]. Different studies confirm that* de novo *lipogenesis might be upregulated in NAFLD [10–12]. Recent reports have shown that endogenous cannabinoids (EC) are related to fatty liver metabolism [13, 14] although the molecular mechanism by which EC modulates the metabolism within hepatocytes is still not clear.

EC are lipid mediators that produce similar effects to those of marijuana by acting on membrane-bound receptors and regulating appetite behaviour [15]. Cannabinoid receptors are mainly localized in the brain, but are also present in small amounts in liver and other peripheral tissue (CB1) and in immune and haematopoietic cells (CB2) [16, 17]. EC may also regulate peripheral energy metabolism, as demonstrated by their CB1-mediated effect on lipoprotein lipase activity in adipocytes [18] and their ability to stimulate lipogenesis in hepatocytes [13, 14]. In agreement with that data, other studies have shown that CB1 receptor antagonists represent an important therapeutic target, owing to beneficial effects on lipid metabolism and in light of its antifibrogenic properties. Unfortunately, the brain-penetrant CB1 antagonist rimonabant was withdrawn because of an alarming adverse effect on mood. However, the efficacy of peripherally-restricted CB1 antagonists with limited brain penetration has now been validated in preclinical models of NAFLD [19].

Taken together, these findings indicate that CB1 receptors mediate metabolic steatogenesis in the liver by central and peripheral effects. Regarding CB2, results of recent studies have suggested that this receptor could be a promising anti-inflammatory and antifibrogenic target [19, 20], although clinical development of its agonists is still awaited.

In order to investigate the associations of CB1 and CB2 with NAFLD we aimed to (1) find out the gene expression profiles of CB1 and CB2 in liver of morbidly obese women with or without NAFLD, (2) assess the relationship between its gene expressions and the presence of hepatic fat and inflammation, and (3) study the relationship between liver CB1 and CB2 mRNA expression and liver mRNA expression of key genes involved in lipid metabolism: genes involved in* de novo* synthesis of fatty acids (ChREBP, SREBP1c, LxR*α*, FxR, ACC1, and FAS), fatty acid oxidation (PPAR*α*), uptake and transport (PPAR*γ*, CD36, and FABP4), and inflammatory related genes (PPAR*δ*, IL6, TNF*α*, and CRP).

## 2. Patients and Methods

### 2.1. Subjects

The institutional review board approved the study. All participants gave written informed consent for participation in medical research. This study included 72 morbidly obese (MO) women (body mass index, BMI > 40 Kg/m^2^) of Western European descent.

We analysed 72 liver samples from MO women. Liver biopsies were obtained during planned bariatric surgery. All biopsies were carried out under clinical indications.

NAFLD was diagnosed by the following criteria: (1) liver pathology, (2) an intake of less than 10 gr of ethanol/day, and (3) appropriate exclusion of other liver diseases. Liver samples were scored by two experienced hepatopathologists using the methods described before [21, 22].

According to their liver pathology, patients were subclassified into the following groups: (1) MO with normal liver (NL) histology (*n* = 16), (2) MO with simple steatosis (SS) (micro/macrovesicular steatosis without inflammation or fibrosis, *n* = 28), and (3) MO with nonalcoholic steatohepatitis (NASH) (Brunt grade 1–3, *n* = 28).

Subjects' weight was stable, with no fluctuation greater than 2% of body weight for at least 3 months prior to surgery. The exclusion criteria were (1) concurrent use of medication known to produce hepatic steatosis, (2) patients using antidiabetics or lipid-lowering medications, including PPAR*α* or -*γ* agonists, (3) diabetic women that were receiving insulin or on medication likely to influence endogenous insulin levels, (4) menopausal and postmenopausal women and subjects receiving contraceptive treatment, and (5) patients who had an acute illness, current evidence of acute or chronic inflammatory or infectious diseases, or end-stage malignant diseases.

### 2.2. Anthropometrical and Biochemical Analysis

A complete anthropometrical examination and a biochemical analysis were carried out on each patient. Height and weight were measured with the patient standing in light clothes and shoeless. BMI was calculated as body weight divided by height squared (kg/m^2^). Laboratory studies included glucose, insulin, glycated haemoglobin, total cholesterol, high-density lipoprotein cholesterol, low-density lipoprotein cholesterol, triglycerides, and transaminases, all of which were analysed using a conventional automated analyser. Insulin resistance (IR) was estimated using homeostasis model assessment of IR (HOMA2-IR) [23]. Serum levels of adiponectin (Linco Research, Inc., St. Charles, USA), resistin (Biovendor, Modrice, Czech Republic), interleukin 6 (IL6) (Quantikine, R&D Systems, Minneapolis, USA), tumour necrosis factor receptor 2 (TNFRII) (AssayPro, St. Charles, USA), and C-reactive protein (CRP) (Dade Behring, Marburg, Germany) were measured in duplicate using ELISA, following the manufacturer's instructions.

### 2.3. RNA Isolation and Real Time PCR

Liver samples were conserved in RNAlater (Sigma, Barcelona, Spain) for 24 hours at 4°C and then stored at −80°C. Total RNA from liver tissue was isolated using the RNeasy mini kit (Qiagen, Barcelona, Spain), according to the manufacturers' protocols. RNA was digested with DNase I (RNase-Free DNase set; Qiagen). First-strand cDNA was synthesized using an equal amount of total RNA with High Capacity RNA-to-cDNA Kit (Applied Biosystems, Madrid, Spain). The Real-time quantitative PCR was carried out in a final volume of 20 *μ*L, which contained 10 ng of reverse-transcribed cDNA, 10 *μ*L of 2X TaqMan Fast Universal PCR Master Mix (Applied Biosystems), and 1 *μ*L TaqMan Assay predesigned by Applied Biosystems for the detection of CB1, CB2, ChREBP, SREBP1c, FxR, LxR*α*, ACC1, FAS, PPAR*α*, PPAR*γ*, CD36, FABP4, PPAR*δ*, IL6, TNF*α*, CRP, adiponectin, and resistin gene and for GAPDH, which was used as the housekeeping gene. The mRNA expression for each gene and sample was calculated using the recommended 2^−ΔΔCt^ method. All reactions were carried out in duplicate in 96-well plates using the 7900HT Fast Real-Time PCR systems (Applied Biosystems).

### 2.4. Statistical Analysis

All the values reported are expressed as mean ± SD (standard deviation) and were analysed using SPSS/PC+ for windows statistical package (version 20.0; SPSS, Chicago, IL). Differences between groups were calculated using the Student's *t*-test or one-way ANOVA analysis. The strength of association between variables was calculated using Pearson's method for parametric variables and Spearman's *ρ*-correlation test for nonparametric contrasts. A multiple linear regression analysis was carried out. The predictors for stepwise linear regression analysis were based on correlation analysis and selected from the variables known to be associated with the dependent variable. *P* values <0.05 were considered to be statistically significant.

## 3. Results

### 3.1. Baseline Characteristics and General Laboratory Data of the Subjects in the Study

Morbidly obese women were classified according to the presence of NAFLD (Table 1). Age, anthropometrical measurements, glucose, HbA1c, insulin, HDL and LDL-Cholesterol, and triglycerides were not significantly different among morbidly obese women in the NL, SS, or NASH groups. However, our results indicated that AST and ALT activity were higher in both the SS and the NASH groups than in obese women with normal liver histology (SS: AST *P* = 0.05, ALT *P* < 0.001; NASH: AST *P* = 0.042, ALT *P* < 0.001). ALP activity was higher in the NASH group than in NL (*P* = 0.032). Moreover, the levels of adipocytokines determined were not significantly different between the morbidly obese with NL, SS, or NASH (Table 1).

### 3.2. Evaluation of CB1 and CB2 mRNA Expression in Liver

**Figure 1:**
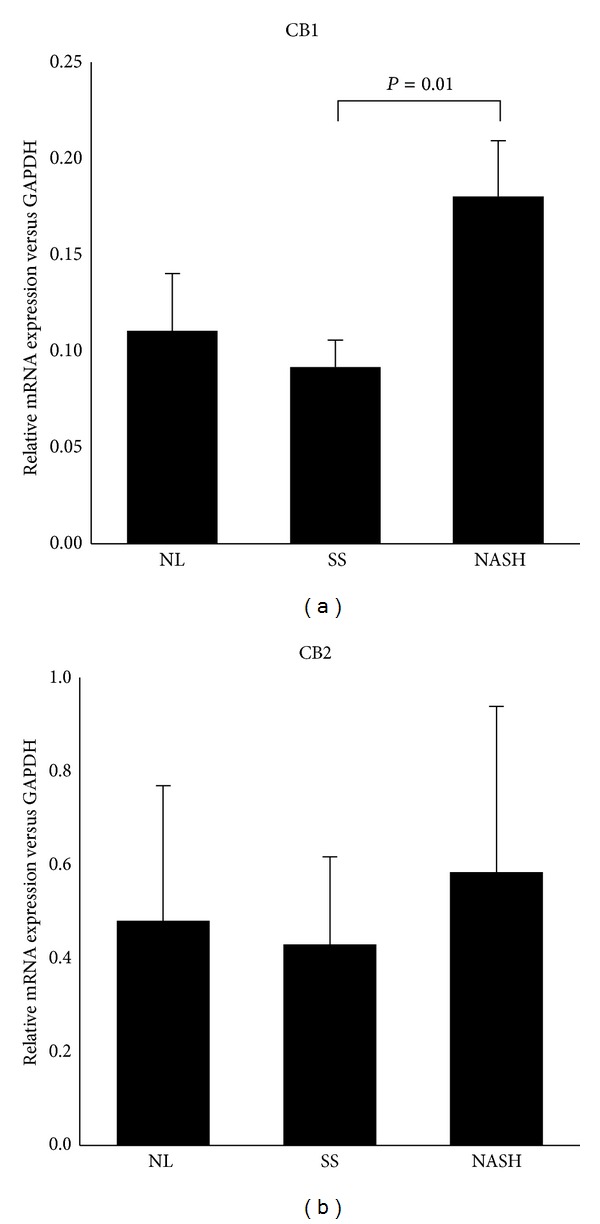
Liver CB1 and CB2 mRNA expression in morbidly obese women classified according to the liver pathology. NL: with normal liver histology; SS: simple steatosis; NASH: nonalcoholic steatohepatitis. Results are shown as mean ± SD. *P* < 0.05 is considered statistically significant.

We analysed hepatic CB1 and CB2 mRNA expression in the MO cohort in relation to the presence of NAFLD. When we subclassified that cohort into NL, SS, and NASH, we observed that CB1 mRNA expression was significantly higher in NASH compared with SS (SS: 0.09 ± 0.07; NASH: 0.14 ± 0.03; *P* < 0.010) (Figure 1(a)). However, CB2 gene expression was similar among the three groups (Figure 1(b)).

Additionally, we studied the relationship between the grade of steatohepatitis and CB1 and CB2 gene expression. When we subclassified the MO cohort into those with NASH Brunt 1 and with NASH Brunt 2/3, we found that both CB1 and CB2 mRNA expression were similar in both groups (data not shown).

### 3.3. Correlations between the Gene Expression of CB1 and CB2 and Genes Related to Fatty Acid Synthesis (ChREBP, SREBP1c, LxR*α*, FxR, ACC1, and FAS), Fatty Acid Oxidation (PPAR*α*), Uptake and Transport (PPAR*γ*, CD36, and FABP4), Inflammation (PPAR*δ*, IL6, TNF*α*, and CRP), and Adipokines (Adiponectin and Resistin) in Liver from MO Cohort

We found a negative correlation between CB1 and PPAR*α* gene expression. We also found that CB2 mRNA expression correlated positively with ACC1 and PPAR*γ* mRNA expression (Table 2).

Regarding inflammation and adipokines, we did not find any correlation between CB1 gene expression and inflammatory genes expression, nor with adipokines expression. However, we found positive correlations between CB2 and IL6, TNF*α*, resistin, and adiponectin geneexpression (Table 2).

In addition, we performed a stepwise multiple linear regression analysis, which included age, BMI, triglycerides, PPAR*α*, and the presence of NASH as independent variables, and CB1 expression as a dependent variable. The results indicated that NASH and PPAR*α* (inverse) were the only variables associated with CB1 expression (*R* ^2^ = 0.255, *P* = 0.002; *R* ^2^ = 0.129, *P* = 0.014, resp.).

## 4. Discussion

The present study demonstrates that in morbidly obese women with NAFLD, liver CB1 gene expression is significantly higher at the histological stage of NASH compared to SS. This finding might agree with experimental studies in obese rats, which showed that rimonabant, a selective CB1 receptor antagonist used as an adjunctive treatment of obesity, reduces liver inflammation [24, 25]. The underlying mechanism has not yet been delineated but in hepatocytes CB1 receptors might contribute to the acute phase response via activation of ChREBP, a liver-specific transcription factor that upregulates acute phase response genes [26]. In our case, we were not able to demonstrate a positive relationship between CB1 and ChREBP mRNA expression, nor with mRNA expression of inflammatory genes.

Studies in cultured hepatocytes and in animal models have observed steatogenic properties of CB1 as a result of hepatic lipogenesis activation, reduction of fatty acid oxidation, and decreased release of TG-rich VLDL, combined to CB1-dependent release of free fatty acids from the adipose tissue [24, 25, 27]. Our cohort did not demonstrate any relationship between the hepatic CB1 gene expression and simple steatosis. However, we did find a negative correlation between CB1 and PPAR*α* gene expression. PPAR*α* plays a pivotal role in fatty acid (FA) catabolism by upregulating the expression of numerous genes involved in mitochondrial FA oxidation, peroxisomal FA oxidation, and other aspects of FA metabolism [28]. Furthermore, PPAR*α* is related to adipoR2. Activation of adipoR2 can increase PPAR*α* levels and activate PPAR*α* pathways, leading to increased fatty acid oxidation and a reduction in oxidative stress [29, 30]. Recently in experimental studies the adipoR agonist (AdipoRon) bound to both adipoR1 and AdipoR2* in vitro*. AdipoRon showed very similar effects to adiponectin in muscle and liver, such as an activation of AMK and PPAR*α* pathways, and ameliorated IR and glucose intolerance in mice fed a high-fat diet, which was completely obliterated in adipoR1 and AdipoR2 double-knockout mice [31]. In conclusion, the higher expression of CB1 in NASH and the negative correlation with PPAR*α* suggest a deleterious role of CB1 in NAFLD.

Regarding hepatic CB2 expression, we did not find any differences between MO with NL, SS or NASH. It is important to note that the role of CB2 in liver diseases is controversial. Some authors have reported that CB2 receptors display protective properties during liver injury. These effects largely depend on anti-inflammatory and antifibrogenic signals generated by CB2-expressing hepatic immune cells and/or hepatic myofibroblasts, with paracrine impact on hepatocytes, which do not express CB2 [19, 32]. The endogenous or exogenous activation of CB2 receptors has been described as a protective pathway in several models of acute liver injury in which CB2 receptors undergo early induction in nonparenchymal cells [16, 20, 33, 34].

We found that CB2 liver expression correlated positively with hepatic ACC1 gene expression, a gene related to fatty acid synthesis. In fatty acid synthesis process, ACC1 converts acetyl-CoA, an essential substrate of fatty acids, to malonyl-CoA. Fatty acids as well as acyl-CoA and acetyl-CoA have been identified as potential causes of lipotoxicity [35]. Furthermore, we found that CB2 liver expression correlated positively with PPAR*γ* gene expression. In NAFLD, PPAR*γ* is upregulated in liver tissue and liver-specific* PPAR*γ** knockout mice are protected from diet-induced steatosis [36, 37]. However, these isolated correlations do not clarify the role of CB2 in lipogenic and uptake and transport pathways.

Other studies provide additional evidence that supports the potential involvement of CB2 receptors in inflammatory liver diseases [38]. However, in our study, we found a positive correlation between liver CB2 and adiponectin gene expression, which is a molecule with an anti-inflammatory role. Taken together, all of this suggests that CB2 seems to be a molecule with a dual action.

Our cohort of morbidly obese women has made it possible to establish clear relationships between NASH and CB1 liver expression without the interference of such confounding factors as gender or age. However, the results of our study cannot be extrapolated to other obesity groups, to men, or to normal-weight subjects.

The main results of our study demonstrate that liver CB1 mRNA expression is induced in nonalcoholic steatohepatitis and correlates negatively with hepatic PPAR*α* expression, which suggests a deleterious role of CB1 in NAFLD. Furthermore, liver CB2 mRNA expression is related to the expression of key genes involved in hepatic lipid metabolism. With regard to inflammation, CB2 seems to act as a dual molecule because it correlates positively with the anti-inflammatory molecule adiponectin and, paradoxically, also with inflammatory genes. Our results suggest that endocannabinoid receptors might be involved in the physiopathological processes of NAFLD and justify the need for further study.

## Figures and Tables

**Table 1:** Characteristics of study cohort classified according to the liver pathology.

	Morbidly Obese (*n* = 72)		
	NL (*n* = 16)	SS (*n* = 28)	NASH (*n* = 28)
	Mean ± SD	Mean ± SD	Mean ± SD
Age (years)	44.0 ± 3.2	47.4 ± 1.5	45.9 ± 1.4
Weight (kg)	121.0 ± 12.1	121.8 ± 16.3	123.8 ± 14.3
WC (cm)	129.5 ± 6.4	132.1 ± 10.7	135.1 ± 9.9
BMI (kg/m^2^)	48.6 ± 2.6	48.1 ± 7.8	47.5 ± 5.4
Glucose (mg/dL)	101.8 ± 18.9	125.4 ± 38.2	119.0 ± 30.0
Insulin (mUI/L)	11.4 ± 3.1	21.2 ± 11.5	23.1 ± 28.1
HbA1c (%)	4.3 ± 0.3	5.9 ± 1.7	5.8 ± 1.7
HOMA2-IR	1.5 ± 0.5	2.9 ± 1.4	3.0 ± 3.4
HDL-C (mg/dL)	43.6 ± 7.6	39.3 ± 9.5	41.5 ± 9.0
LDL-C (mg/dL)	102.0 ± 31.7	104.7 ± 24.6	102.5 ± 32.6
Triglycerides (mg/dL)	144.4 ± 51.9	178.4 ± 68.5	183.1 ± 86.6
AST (U/L)	25.8 ± 8.5	43.7 ± 34.4*	44.9 ± 29.3*
ALT (U/L)	25.4 ± 10.5	45.6 ± 31.6*	46.3 ± 28.3*
GGT (U/L)	18.3 ± 13.8	30.6 ± 22.2	36.1 ± 31.7
ALP (U/L)	58.2 ± 12.6	66.3 ± 16.0	71.9 ± 15.2*
Adipocytokine levels			
Adiponectin (µg/mL)	10.1 ± 2.0	6.8 ± 3.5	6.7 ± 2.4
IL6 (pg/mL)	2.1 ± 0.6	2.9 ± 2.5	3.3 ± 2.0
Resistin (ng/mL)	5.1 ± 2.1	4.6 ± 2.0	4.5 ± 1.6
TNFRII (ng/mL)	4.2 ± 1.4	5.5 ± 2.5	5.6 ± 1.7
CRP (mg/dL)	1.0 ± 0.8	0.9 ± 0.7	1.1 ± 0.7

NL: morbidly obese subjects with normal liver; SS: morbidly obese subjects with simple steatosis; NASH: morbidly obese subjects with steatohepatitis; ALT: alanine aminotransferase; ALP: alkaline phosphatase; AST: aspartate aminotransferase; BMI: body mass index; CRP: C-reactive protein; GGT: gamma-glutamyltransferase; HbA1c: glycosylated haemoglobin; HDL-C: high density lipoprotein; HOMA2-IR: homeostatic model assessment 2-insulin resistance; IL6: interleukin 6; LDL-C: low density lipoprotein; TNFRII: tumour necrosis factor receptor II; WC: waist circumference. *indicates significant differences respect NL group (*P* < 0.05).

**Table 2:** Correlations between the expression of CB1 and CB2 and genes related to fatty acid synthesis, to fatty acid oxidation, uptake and transport, inflammation, and adipokines in liver from morbidly obese women.

	CB1		CB2	
	*r*	*P* value	*r*	*P* value
*De novo* synthesis of Fatty acids				
ChREBP	0.185	0.198	0.072	0.603
SREBP1c	0.074	0.590	0.031	0.816
LxR*α*	0.052	0.709	0.126	0.346
FxR	0.079	0.571	0.095	0.482
ACC1	0.207	0.126	0.257	**0.047**
FAS	0.164	0.232	0.182	0.167
Fatty acid oxidation				
PPAR*α*	−0.354	**0.037**	0.155	0.347
Fatty acid uptake and transport				
PPAR*γ*	0.161	0.240	0.600	**<0.001**
CD36	−0.028	0.846	0.157	0.257
FABP4	0.105	0.460	0.040	0.776
Inflammation				
IL6	0.116	0.431	0.288	**0.040**
TNF*α*	−0.018	0.902	0.293	**0.033**
CRP	−0.210	0.140	−0.002	0.988
PPAR*δ*	−0.077	0.576	0.126	0.342
Adipokines				
Adiponectin	0.031	0.846	0.625	**<0.001**
Resistin	−0.058	0.701	0.506	**<0.001**

*P* < 0.05 are considered statistically significant.

## List of Abbreviations

ACC1: Acetyl-coenzyme A carboxylase 1
ALT: Alanine aminotransaminase
AST: Aspartate aminotransaminase
ALP: Alkaline phosphatase
BMI: Body mass index
CB1: Cannabinoid receptor 1
CB2: Cannabinoid receptor 2
CD36: Fatty acid translocase
ChREBP: Carbohydrate response element binding protein
CRP: C-reactive protein
EC: Endogenous cannabinoids
FABP4: Fatty acid binding protein 4
FAS: Fatty acid synthase
FxR: Farnesoid X receptor
GGT: *γ*-glutamyl transferase
HbA1c: Glycosylated hemoglobin
HDL-C: High-density lipoprotein
HOMA2-IR: Homeostatic model assessment method insulin resistance
IL6: Interleukin 6
IR: Insulin resistance
LDL-C: Low-density lipoprotein
LxR*α*: Liver X receptor
NAFLD: Nonalcoholic fatty liver disease
NASH: Nonalcoholic steatohepatitis
NL: Normal liver
PPAR*α*: Peroxisome-proliferator-activated receptor *α*
PPAR*δ*: Peroxisome-proliferator-activated receptor *δ*
PPAR*γ*: Peroxisome-proliferator-activated receptor *γ*
SS: Simple steatosis
SREBP1c: Sterol-regulatory-element-binding protein
TNF*α*: Tumour necrosis factor
VLDL: Very low-density lipoprotein
WC: Waist circumference.
